# Impacts of road development in sub-Saharan Africa: A call for holistic perspectives in research and policy

**DOI:** 10.1016/j.isci.2025.111913

**Published:** 2025-01-28

**Authors:** Lisa Biber-Freudenberger, Christina Bogner, Georg Bareth, Michael Bollig, Peter Dannenberg, Javier Revilla Diez, Clemens Greiner, Philipo Jacob Mtweve, Britta Klagge, Tanja Kramm, Detlef Müller-Mahn, Vincent Moseti, Nicodemus Nyamari, Dennis Otieno Ochuodho, Elias Kuntashula, Theobald Theodory, Jessica Paula Rose Thorn, Jan Börner

**Affiliations:** 1 Center for Development Research, University of Bonn, 53113 Bonn, Germany; 2 Ecosystem Research, Institute of Geography, Faculty of Mathematics and Natural Sciences, University of Cologne, 50674 Cologne, Germany; 3 Global South Studies Center, University of Cologne, 50931 Cologne, Germany; 4 GIS and Remote Sensing Group, Institute of Geography, University of Cologne, Otto-Fischer-Straße 4, 50674 Cologne, Germany; 5 Department of Geography, University of Bonn, 53115 Bonn, Germany; 6 School of Biological and Physical Sciences, Jaramogi Oginga Odinga University of Science & Technology, Bondo, Kenya; 7 Department of Agricultural Economics and Extension Education, University of Zambia, Lusaka, Zambia; 8 Department of Environment and Sustainable Development, Mzumbe University, Mzumbe, Tanzania; 9 Centre for Environmental Policy, Imperial College London, Weeks Building, 16-18 Prince’s Gardens, London SW7 1NE, UK; 10 Department of Environmental Sciences, University of Namibia, Private Bag Windhoek 13301, Namibia; 11 Institute for Food and Resource Economics, University of Bonn, 53113 Bonn, Germany

**Keywords:** Human geography, Environmental science, Nature conservation, Human Geography

## Abstract

This perspective explores the multifaceted development challenges related to road network expansion in sub-Saharan Africa, where recent infrastructure investments reflect transformative ambitions but also imply socio-ecological tradeoffs. Roads can boost economic growth by facilitating trade, tourism, and access to essential services, yet they simultaneously contribute to ecosystem fragmentation, biodiversity loss, and human-wildlife conflicts. Looking at the history of Africa’s road development, we find that mega-projects—often funded by international donors—reshape political and economic landscapes while altering rural livelihoods and ecosystems. We synthesize literature and case studies to reveal critical trends and propose solutions, urging for a shift toward sustainable, evidence-based infrastructure strategies that balance development with environmental stewardship. We further advocate for transdisciplinary approaches and community engagement to align road expansion with long-term stakeholder needs so as to minimize adverse impacts on Africa’s socio-ecological systems.

## Graphical abstract

**Figure:**
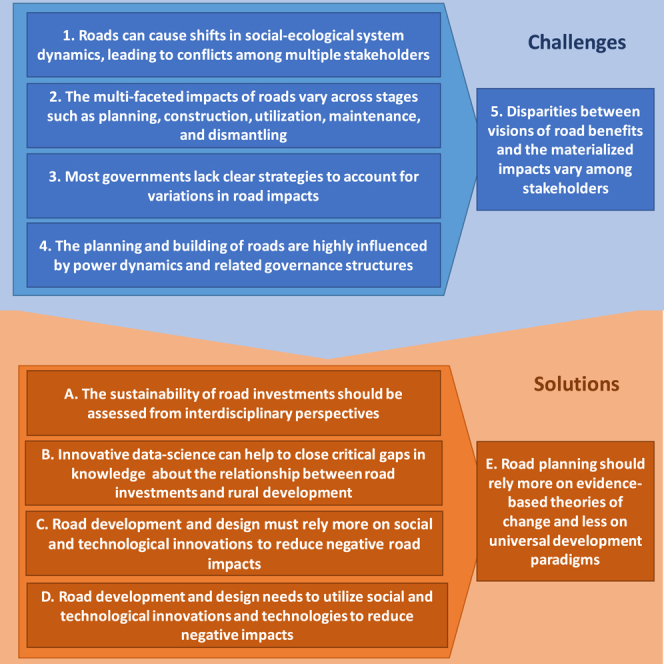

## Introduction

In recent years, the Global South, especially sub-Saharan Africa, has witnessed a surge in road infrastructure development. This growth has been fueled by significant investments into the development of roads and highways, signaling an era of progress, modernity and connectivity.^1^ In sub-Saharan Africa, road infrastructure presently remains the means of conveying about 75% of freight and passengers.^2^ The expansion of road networks is often suggested as a major step toward modernity, with a promise of improved connectivity and economic progress.^3^ Indeed, many studies have shown that roads play an important role for the economic development in different regions,^4^ such as through facilitating trade and transport of goods,^5^ boosting tourism^5^ and providing access to physical amenities (e.g., schools, hospitals, markets), technical infrastructure (e.g., power grids, communication, dams), and social infrastructure (e.g., education and healthcare services^6^).

In Africa, road perceptions and visions have evolved across four distinct epochs—precolonial, colonial, post-colonial, and contemporary. Pre-colonial Africa had developed a sophisticated, long-distance trade network involving caravans trading ivory and other goods and relying heavily on professional porters.^7^ During the colonial period, this existing infrastructure was reshaped and often replaced by paved roads to allow a more effective extraction of natural resources and their trade.^7^^,^^8^ In the 1950s and 1960s, a transitional phase between colonial and post-colonial periods, international loans, including from the World Bank, went primarily into road infrastructure.^9^ However, limited economic growth, stagnating rural development and a lack of foreign direct investments, led to a general disillusionment about the potential and promises of public road management.^9^^,^^10^

The post-colonial period was marked by efforts of new governments to meet the expectations of an increasing population and to address infrastructure deficits inherited from the colonial period. While resource extraction was the main goal of infrastructure development during the colonial period, increasing connection between communities and social infrastructure, became a higher priority post-independence. Despite limited financial resources, some countries (e.g., Kenya) were able to make substantial public investments and to expand road infrastructure connecting rural areas with each other.^11^

Since the 1980s, more reforms have taken place to support structural adjustments including a stronger focus on road maintenance and rehabilitation and facilitating transitioning to integration efforts linking regions globally.^11^ Currently, infrastructure investments are enjoying a comeback with 79 development corridors being either underway or planned across the continent.^12^ The current period is marked by large projects including new actors and developers (e.g., public-private partnerships) and new growth paradigms (e.g., value chain creation^13^). But, as in previous periods, the local reality continues to be characterized by informal processes of use, development, and growth (e.g., street markets^14^).

**Figure 1:**
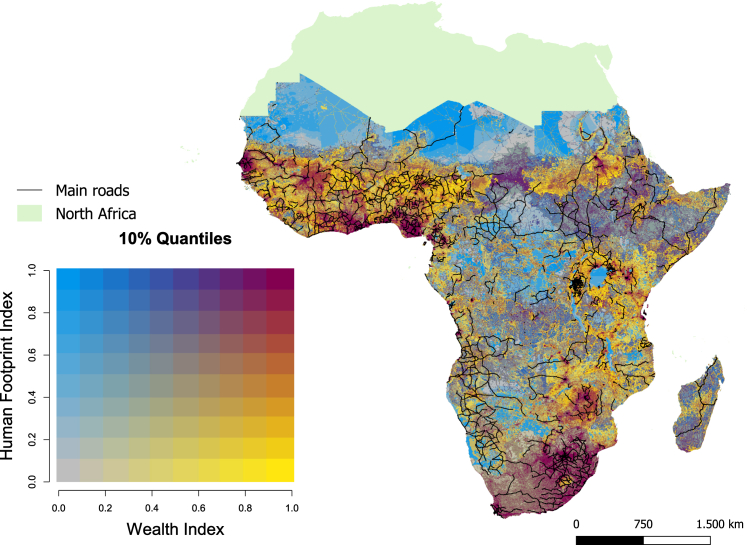
Roads, wealth, and human footprint Bivariate map showing Wealth Index^21^ and Human Footprint Index^22^ data together with main roads.^23^

The combination of these old and new processes leads to new spatial configurations and unintended positive and negative outcomes of roads on ecosystems, rural livelihoods as well as the interaction between these dimensions.^15^^,^^16^^,^^17^ While extensive research has explored the impacts of roads on ecosystems, species, and livelihoods individually, there remains a notable scarcity of studies addressing their interplay.^18^ Roads intersect diverse landscapes, fragmenting habitats and disrupting ecological functions. As a result, studies have found an increasing loss of biodiversity, altered migration patterns, nature resource degradation through air, soil, and water pollution and increased human-wildlife conflicts.^19^^,^^20^ These ramifications extend beyond the immediate vicinity of the roads, influencing adjacent ecosystems and rural or peri-urban communities.^19^ Overlay analyses reveal that areas with high road densities are typically found where high human footprint indices align with high wealth index values. (Figure 1).

Furthermore, the impact of roads on local livelihoods is multifaceted. Studies have shown that the introduction of roads can trigger large scale shifts in traditional livelihood strategies.^20^ New economic avenues may emerge, but the disruption of established practices can also lead to vulnerabilities, particularly of marginalized communities, such as sociocultural changes, land acquisition and displacement, increasing the cost of living due to higher land prices, spread of diseases, widening social inequality, and safety concerns.^17^^,^^24^^,^^25^

Moreover, the trajectory of road development is profoundly intertwined with the broader geopolitical context. International donors often play a pivotal role in offering loans, grants or concessional financing to build or upgrade these projects, influencing not only the physical layout of the roads but also the underlying political and economic structures.^26^^,^^27^ Donors can positively influence governance, the application of international environmental and social safeguards, policy reforms, and accountability.^28^ Still, the accumulation of foreign debt and repayment obligations, strains on national budgets, asset capture, and overreliance on single creditor also implies comes with risks.^29^ Uncritical support to infrastructure investments may thus silently shift decision-making power in favor of political and financial interests and weaken evidence-based and participatory governance mechanisms.

**Figure 2:**
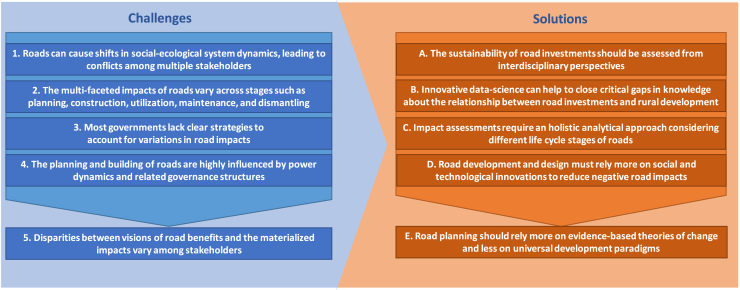
Challenges and solutions Overview of challenges and their potential solutions for science and policy in the context of road development and associated investments.

In the following, we first explore challenges for the scientific research and decision making around road development derived from a nuanced understanding of the complex interactions between roads, societies, and ecosystems, uncovering critical trends and patterns that demand careful consideration. Then we suggest potential solutions to tackle these challenges and suggest directions for further development of research, planning and governance of road development (Figure 2).

### Roads can cause shifts in social-ecological system dynamics, leading to conflicts among multiple stakeholders

Road infrastructure plays a pivotal role in economic growth, enhancing communication and technology, creating job opportunities^26^^,^^30^ and enhancing mobility and connectivity.^31^^,^^32^ However, urban sprawl and the expansion of agricultural land^33^ as well as the increasing establishment of roadside hotels, restaurants, business opportunities for taxi drivers,^34^^,^^35^ and the concentration of transport opportunities can also have negative socioeconomic impacts, which may vary over space and time and between different contexts (cf. challenges no. 2 and 3).

Roads planned or already built affect land value^26^ and can drive land speculation leading to the gradual displacement of poorer community members, small-scale and traditional farming as well as indigenous communities^33^^,^^36^ causing additional land disputes and conflicts.^37^ In Kenya, Greiner et al.^17^ found, for example, that people living closer to roads are characterized by more diversified livelihoods (i.e., combining different income opportunities) and higher education but are also more likely to be affected by land use conflicts. While in the Global South positive and negative outcomes of roads are highly spread between different groups and individuals, they typically depend on information access to the planning process (e.g., to get an early mover advantage) landownership and financial capacities, but often also on cronyism and corruption^38^ (cf. challenge no. 4). Through people’s economic differences in ceasing the opportunity brought about by increased accessibility, poverty and vulnerability may be aggravated (cf. challenge no. 5).

Roads have substantial negative impacts on biodiversity through increased habitat fragmentation, deforestation, land degradation, pollution, poaching, road kills and spread of invasive species.^26^ Roads effects often unfold in a stepwise manner as they spawn networks of secondary and tertiary roads that subsequently improve the access to previously undisturbed areas^39^ leading to cascading and interlinked effects on ecosystems and human wellbeing.^40^^,^^41^ Deforestation for road construction increases, for example, carbon emissions, reduces the cooling effect of plants, alters wind patterns and local microclimates, causes erosion and increased sedimentation in streams.^29^

Air, water, and soil pollution due to emissions by vehicles, microplastic from tire abrasion and problematic polymers and heavy metal in asphalt can be harmful to natural ecosystems and human health.^42^^,^^43^ Unpaved roads increase the breeding of vectors of pathogens and diseases, especially in pools of stagnant water in the ditches, potholes, and or unflattened soil mounds. Vehicle emissions significantly contribute to air pollution and dust rising from unpaved roads can lead to airborne diseases and respiratory illnesses.

Furthermore, roads alter water flows and sedimentation, consequently affecting aquatic ecosystems.^44^ Roads also act as pathways for the spread of invasive plant species, some of which are associated with an increase in disease vectors such as mosquitoes.^45^ They also create substantial barriers to wildlife movement, e.g., in the Serengeti,^46^ and drive human-wildlife conflicts pushing wildlife into neighboring communities.^47^ Conclusively, research has shown that roads cause short and long-term impacts depending on the viewing perspective, different stakeholder groups, spatial and temporal scales and the life cycle stage of a road (cf. challenge no. 2). The positive impacts often come with trade-offs, and short-term effects may gradually spill over to cause irreversible damages.

### The multi-faceted impacts of roads often vary across stages such as planning, construction, utilization, maintenance, and dismantling

While most studies outlined in the previous section (cf. challenge no. 1) focus on the impacts of roads during their usage and maintenance phases, it is crucial to recognize that varying impacts on society and ecosystems also emerge and may fluctuate across other stages of road development.^48^ These impacts are not only evident after road development but can manifest long before construction begins, during planning stage, or as soon as knowledge of the plans is available.^49^ First, the information may be accessible to selected individuals such as development planners, government administrators, investors, and developers granting them unjust advantages, including opportunities for new businesses and land speculation. However, this contrasts sharply with the experiences of vulnerable community members, who are often excluded from such information and associated benefits (cf. challenges no. 4 and 5). These phenomena were for example demonstrated by Kinuthia et al.^49^ in the construction of Eastern bypass in Nairobi, Metropolitan, Kenya that significantly increased the land subdivision and transactions among different groups in the bypass vicinity.

Road construction requires space and workforce. However, creating space for road corridors frequently results in human relocations, evictions, demolitions, and disruptions to livelihoods and the ecosystems.^50^ These impacts manifest in human and property rights violations, community disintegration, pollution, deforestation, and land degradation due to use of heavy machinery.^35^ While construction generates job opportunities, concerns arise regarding labor conditions, especially for young workers.^51^ For instance, the 2022 strike by construction workers on the Gibeon-Tses road project in Namibia highlighted demand for improved working conditions such as salaries, job security, accommodation, and adherence to potable water safety guidelines.^52^

Historically, many roads have progressively evolved from footpaths to dirt roads and later to tarmac roads depending on the growing demands for the upgrade. However, in the recent past a number of roads have been built for specific purposes, e.g., to serve as feeder roads to major trunks or as “ancillary infrastructure” for renewable energy projects such as geothermal.^53^ Throughout their lifetime, roads can be repurposed to other uses. For instance, roads built for local communities’ use may be repurposed to provide access to protected areas for tourism. Changing the purposes of the roads can have both positive and negative impacts on different stakeholders triggering conflicts (cf. challenges no. 1 and 5). This can be exemplified by the changing purpose of roads in the Serengeti ecosystem in Tanzania. In the early 1950s, the roads were used by rural communities; however, the ecosystem was gazetted as protected area, thus restricting local communities from using it. Communities would typically use these roads, but transit fees make them unaffordable for poorer members. For example, a fee of 11,800 Tshs (approximately $4.7) is levied to use the road through Serengeti National Park^54^; or Ngorongoro Conservation Area.^55^

Even if some roads are abandoned or dismantled after their use phase, their impacts still persist beyond their lifetime. Additionally, the time for restoring the ecosystems from negative road impacts such as pollution, land degradation and biodiversity loss, are highly disproportionate to the actual lifespan of roads as these former roads often continue to be used for illegal resource exploitation.^56^^,^^57^

### Most governments lack clear strategies to account for variations in road impacts

The outlined road impacts (cf. challenges no. 1 and 2) also depend on interactions between the road type and the geographic context.^58^ The type of road can be broadly defined by its purpose (e.g., highway or feeder road) with certain implications for its surface (paved or unpaved), the width (one lane or multiple lanes), and the amount and type of traffic on that road (transport of goods and/or people, tourist traffic, etc.).

In the African context, paved roads are considered as all-weather roads as they are less affected by heavy rains than dirt roads, reduce transportation costs as well as post-harvest losses, and provide better access, e.g., to health services. However, while some research suggests that roads with high traffic and more capacity for cargo and people (e.g., highways) have a stronger economic effect on livelihoods compared to smaller feeder roads, other studies find the opposite.^59^^,^^60^

Paved roads and associated vehicular traffic, especially in high biodiversity areas, affect wildlife, e.g., through noise and disruption of migratory routes of species. While some species are more likely to be influenced by the land use changes caused by roads, other species are more likely to be prone to road kills or road avoidance if traffic density is high.^61^ Studies have shown that the rate of deforestation and other illegal activities such as wildlife poaching, logging, and forest encroachment differs with road types.^20^

Also deforestation rates have been shown to vary based on the type and width of the roads—with wider roads having a bigger buffer of impact as compared to narrow roads.^62^ The impacts are usually the greatest closer to the road and diminish gradually with increasing distance from the road.^63^ Similarly, the road impact zone for fauna also differs depending on the type of species. For instance, the impact on bird populations is most significant within 1 km, while for mammals the impact extends up to 5 km.^64^

Other studies have shown that feeder roads, especially narrow tracks branching from main paved or unpaved roads can exacerbate deforestation as they are difficult to maneuver hence limiting surveillance. However, in many cases roads are being built in a stepwise process, where unpaved roads become paved roads often with many years in-between. In these cases, it has been shown that road pavement has relatively little additional effects on deforestation.^65^

Similarly, whether feeder roads, secondary roads, or highways have a larger impact on economic benefits and livelihoods is highly context dependent and variable. Sub-Saharan Africa has some of the highest rates of road traffic injuries and deaths in the world,^66^^,^^67^ particularly among the younger population.^68^ Both paved and unpaved roads are prone to road accidents in different ways as newly paved roads see more accidents attributed to increased vehicular traffic, especially where there are multiple road users such as moving livestock and pedestrians. Better road infrastructure has, for example, seen a surge in motorbikes as a cheap mode of transport in most countries of the global south, but they are the leading cause of fatal accidents. An increase in traffic flow may become dangerous for children and elderly with slower reaction times, and the wider community members to socialize and carry out daily activities.^36^

Poor road conditions as we often find in smaller and feeder roads, however, increase the wear and breakage of vehicles and pose major risks for fatalities or serious injuries, also due to vehicle breakdown posing a threat to other users, especially at night. Black spots, i.e., locations that have a history of high number of traffic accidents, are often located in areas with sharp curves, inadequate signage, lack of proper lighting, or poor road surface conditions including potholes.^69^

Although social, economic, and environmental impacts of roads vary not only by type and geographical context, most governments do not follow transparent strategies for the consideration of these impacts in the development of roads. Some authors have argued that this is also due to the political role of road development in the African context^70^ (cf. challenge no. 4).

### The planning and building of roads are highly influenced by power dynamics and related governance structures

The view that the decisions on road allocations are based on economic efficiency and social equity principles has recently been challenged by an alternative perspective that emphasizes political motivations for public investments.^71^^,^^72^ This new perspective is based on the idea that public goods are allocated by politicians, who can be self-interested and susceptible to rent-seeking and corruption.^73^ As one of the most visible public goods, roads feature prominently in political promises, especially targeted to rural dwellers. And, since politics and public goods allocations are geographical in nature, roads have often been geographically and politically allocated to reward supporters and expand political support bases.^74^

Roads are often allocated to specific geographical regions or culturally constructed groups, such as ethnicities. For example, Burgess et al.^75^ found that from 1963 to 2011, Kenyan presidents tended to allocate more roads to districts with co-ethnic majority. This finding aligns with Jablonski’s study^76^ on ethnic favouritism in aid-distribution in Kenya. On a cross-national scale, Graff^77^ showed that the home regions of national African leaders had significantly more infrastructure than the respective average national stock. Furthermore, the literature on the political budget cycle (PBC) demonstrates that politicians manipulate fiscal policies on road development along electoral cycles with expansionary spending in the lead-up to elections.^78^ When comparing such distributive politics among polities, most political economy literature suggests that this behavior is common in authoritarian regimes or weak democracies of developing countries.^74^ However, this general assertion has been contested by others, such as Tavits,^79^ who have shown this conduct to be widespread.

The high financial stakes and political power play in road infrastructure development fuel a self-perpetuating cycle of corruption, hindering effective road development. Corruption is often rife in settings where the political elites collude with contractors to subvert proper procurement procedures and win over-priced government tenders.^80^ Furthermore, investments in mega-transport infrastructure across Africa are criticized as being driven primarily by the greed of local politicians in collusion with multinationals to extract natural resources with little or no benefits to the locals.^39^ While infrastructure investments should support capacity building and rural development, corrupt and politically motivated infrastructure spending often wastes public funds, creates economic distortions, and leaves countries heavily indebted. Since road development is capital intensive, most projects in developing countries are hinged on external funding, engendering external fiscal dependency.^35^ Although public-private partnerships can mitigate this, inadequately structured partnerships may result in prolonged financial liabilities, especially when the anticipated economic benefits, such as enhanced trade (cf. challenge no. 1), do not materialize as soon as expected and not for every stakeholder (cf. challenge no. 5).

### Disparities between visions of road benefits and the materialized impacts vary among stakeholders

Road construction projects in Africa are strongly influenced by visions of regional development, based on better connections of rural areas to urban centers.^24^ In this sense, roads serve as tools of future-making in two ways. They are at the same time life-lines of trade and development, and symbols of modernization. In other words, the construction of roads combines a material and a symbolic dimension: kilometers of tarmac, and visions of a better future. Yet, the material achievements of roads do not always coincide with the visions of the original development plans (cf. challenge no. 2). This deviation between visions and materialized impacts of roads raises the questions which vision of modernity, whose interest is driving road construction and which stakeholders are influencing these projects (cf. challenge no. 4).

All across the African continent roads are being planned and built as the essential elements of development corridors, which aim at connecting peripheral areas to urban growth poles in order to foster investments and economic growth (cf. challenge no. 1). Development corridors have become dominant blueprints for spatial development by copying models of regional integration from middle and high-income areas of the world. Like other tools of spatial planning, the design of corridors simultaneously projects visions into the future and into space. They represent images of desirable futures shaped by various stakeholders. Development corridors may thus be understood as “dreamscapes of modernity.”^1^^,^^81^ Modernity in this context is meant as a copy of dynamically developing regions in Europe or East Asia.

One of the visions of development corridors is the improvement of transboundary integration. The first of these transboundary development corridors implemented in Africa was the Maputo-Johannesburg corridor, which was opened right after the end of Apartheid and the end of the Mozambican civil war in the early 1990s.^82^ This successful model was later adopted for other programs of regional integration. A prominent example is the Lamu Port-South Sudan-Ethiopia (LAPSSET) corridor, which is meant to connect two neighboring land-locked countries of Kenya to the Indian Ocean. In reality, the LAPSSET corridor so far does not so much serve the regional integration promised in its title, but rather the opening up and political control of the arid and semi-arid lands of Northern Kenya with distinct impacts on different stakeholders (cf. challenge no. 1).

Mega-projects like the LAPSSET corridor in Kenya or the Southern Agricultural Growth Corridor of Tanzania (SAGCOT) hardly ever get implemented as originally planned. Even if the original vision that initially inspired the project gets lost or adjusted during the long course of implementation (cf. challenge no. 2), it would probably be too simple to reduce the diagnosis to false promises or failure. Deviation from original plans is partly due also to the long phase of political negotiations, conflicts, and public debates^83^ (cf. challenge no. 4).

Based on these outlined scientific facts, we distill a number of potential solutions for research and decision making in the context of road development.

#### The sustainability of road investments should be assessed from interdisciplinary perspectives, integrating knowledge from social science, humanities, economics, and ecology

Several studies have been conducted to assess the impacts of roads^84^ (cf. challenges no. 1, 2, and 3). Most of these studies either assess the effects of roads on the environment or on certain socioeconomic variables, while very few assess both.^85^

By focusing on only one dimension of road impacts, studies limit their scope to specific disciplinary traditions and methodological approaches that might bias the findings by looking at issues uni-dimensionally, hence failing to offer a holistic view on road impacts. Roads exist within a complex socio-ecological context and their effects are multidimensional.^86^ While acknowledging the complexity of socio-ecological systems, attempting to isolate individual components such as roads for analysis typically falls short of capturing the inherent intricacies.^87^

Roads function as an interface for the interactions between human societies and natural environments. Social science provides insights into human behavior, cultural and historical dynamics, and societal relations as influenced by road networks. Ecology delves into the intricate web of relationships between roads and the environment, highlighting issues like habitat fragmentation/loss, biodiversity loss, and ecosystem disruption. Integrating these knowledge domains is crucial to unraveling the nuanced interactions that shape various road impacts.

To fully understand the intertwined road impacts, there is a need for an interdisciplinary perspective that integrates knowledge from social sciences, humanities, economics, and ecology.^18^ This would require borrowing from different disciplines to take advantage of tools that can be combined to accurately identify areas of optimum social, environmental and economic potential and minimize harmful impacts. For example, many recent studies emanating from development studies and economics fields are concerned with establishing the causal relationship between road investments and consequent socioeconomic outcomes.^88^^,^^89^^,^^90^ The mixed and ambiguous study results from these strands of research have often been attributed to a lack of standard methodologies and the issue of endogeneity and reverse causality that is associated with road placement. Embracing a complexity science approach acknowledges that roads cannot be understood solely by studying their components in isolation. Instead, it recognizes that roads are nodes in a vast network of interdependencies and feedback loops, where changes in one area ripple through the entire system.

Moreover, complexity science provides tools to model and predict non-linear outcomes^91^ and is adept at handling uncertainty and emergent behaviors,^92^ which are common in large-scale infrastructure projects. Additionally, complexity science promotes adaptive and flexible planning approaches.^93^ It aids in evaluating impacts over extended periods and across different scales.^94^ This necessitates interdisciplinary collaboration to explore how roads impact various aspects of society, equity, economics, and ecology, leading to a more holistic assessment but might be limited also by a lack of available data (cf. solution B).

#### Innovative data-science can help to close critical gaps in knowledge about the relationship between road investments and rural development

High-quality datasets are crucial for the assessment of road impacts in different regions and over time. This is true for road data (road location, type, and construction time) but also for data describing road impacts (environmental and social data, challenges 1, 2, and 3).

The use of low-quality datasets can significantly affect the results of analyses and lead to misinterpretations. African countries often suffer in particular from a lack of detailed, disaggregated road data that can be systematically compared over space and time. Information on demarcated boundaries, titles, stages of evolution, and duration of road developments is limited and often comes from various places and sources.^12^ This leads to a lack of standardized file formats and metadata, as well as incomplete and inaccurate datasets. Many available road data are outdated due to the high cost of acquiring and updating it.^95^^,^^96^ To address this issue, openly sourced road data, such as Open Street Maps or gROADS, are often used. However, these datasets are also often only partially up-to-date and of varying quality, as mapping activities in African countries are often spatially limited to areas that were affected by a specific crisis.^96^^,^^97^^,^^98^ Furthermore, they do not provide information about the time when a road was constructed, upgraded, or dismantled, limiting the possibilities for historical and time-series analyses.

To address the scarcity of road data in Africa, road information can be extracted from remotely sensed imagery. Available methods have evolved from manual to various automatic learning-based methods like deep learning, which highly automates the process for modern high-resolution satellite imagery.^99^ Advanced artificial intelligence (AI) techniques can tailor algorithms for varying data quality and formats. Collaborations with international space agencies and private satellite firms can also enhance data access. Moreover, crowdsourced mapping can help to maintain datasets. Efforts are still needed to encourage collaboration for unified data sharing platforms, standardized (meta)data and to resolve legal issues in public data provision. Capacity building can enhance local GIS data expertise and securing dedicated funding for the acquisition and maintenance of road data.

However, historic road data extraction remains challenging due to the lack of suitable historical imagery with appropriate spatial resolution for African countries.^12^ For example, the automatic extraction from historical CORONA imagery is a challenging task due to missing spectral variability of different surface types.^100^^,^^101^ However, recent advancements propose extracting roads from historical topographical maps using advanced deep learning techniques showed promising results for this issue.^102^^,^^103^^,^^104^ These data can be used for different purposes but overall contribute to a more holistic assessment of road impacts (cf. challenges no. 1, 2, and 3 and solution C).

#### Road impact assessments require a holistic analytical approach considering different life cycle stages of roads

Across the world Environmental Impact Assessments (EIAs) are legally mandatory in most countries for large-scale road development projects^105^ and are also increasingly capturing social impacts as part of integrated environmental and social impact assessments (ESIAs).^106^ These assessments are important policy instruments but have faced criticism due to challenges in local capacity to conduct them and implement their results.^107^^,^^108^ Although many ESIAs regulations require the involvement of stakeholders in the assessment process, it is often quite intransparent when and to which extent this involvement is happening.^108^ The effects of roads constructed as ancillary infrastructure for large-scale projects, e.g., in the field of renewable energies in rural areas, are also generally not sufficiently taken into account in impact assessments.^53^

While impact assessments are conducted *ex ante*, most scientific studies on the impacts of roads published in peer-reviewed papers are conducted *ex-post*. While this makes sense on tracking whether and to which extent certain investments have yielded expected economic gains and socioeconomic benefits, this approach is considered reactive, especially if the impacts are negative and irreversible.^109^

Furthermore, neither EISAs nor scientific ex-post assessments usually consider impacts of roads during all life stages (cf. challenge no. 2). As road impacts do not only vary by type and context (cf. challenge no. 3) but also over time, we suggest that a life cycle assessment (LCA) approach is necessary to evaluate road-related impacts.

Road impacts are for example likely to differ during the construction, use, maintenance, and rehabilitation phase (cf. challenge no. 2). Impact assessments usually only consider impacts during the usage phase but not the raw materials, nor the machinery necessary for road construction. Incorporating the long-term costs of maintenance and operation into the initial project planning and budgeting ensures a more holistic view of the project’s status. Other authors have pointed out the need for such an LCA, of which some have been conducted already, but according to multiple authors^110^^,^^111^^,^^112^ none in Africa so far. Apart from their highly inconsistent methodological approach,^110^ most of these studies do not consider the whole life cycle of roads in a cradle-to-cradle manner.^112^^,^^113^

LCA studies in the context of roads have so far focused on the construction phase and the materials being used as well as the direct impacts on climate change but neglected other phases e.g., the maintenance phase^113^ and other impacts on land use change and natural ecosystems.^111^ Furthermore, no LCA study to our knowledge has considered socioeconomic impacts, which would be relevant to assess the costs and benefits of road development in a decision-making context.^111^ Therefore, we suggest that an anticipatory and holistic assessment of road impacts requires methodological developments to incorporate road benefits and costs but also long-term and larger-scale impacts across all life cycle stages. This will also facilitate the development and identification of appropriate social and technological innovations to reduce negative road impacts as much as possible (cf. solution D).

#### Road development and design must rely more on social and technological innovations to reduce negative road impacts

Negative impacts of roads (cf. challenges no. 1, 2, and 3) have led to the development of a large number of different mitigation strategies to reduce them.^16^ These strategies range from better road planning, to technological and social innovations reducing negative impacts of existing roads. It has however also been pointed out that many of the road safety standards were developed based on research on road safety measures in high income countries, making additional research representative of the Global South highly necessary.^114^

Road planning could for example minimize the disturbance of ecosystems, as well as security risks for local communities and businesses by realigning highways away from ecologically sensitive areas, indigenous communities and arable community lands (which however also reduces potential positive effects of connectivity) as well as improving road design.^114^

Alternatively, initiatives could focus on improving existing infrastructure (e.g., fixing road potholes) and creating alternative access points for businesses affected by the road developments. In addition to economic compensation, training, grants, or loans to establish new businesses can be beneficial. Skill development programs, microfinance schemes, and promoting new market opportunities can support alternative livelihoods. Further alternative options in improving the connectivity of people and businesses in remote areas can also include the improvement of digital connectivity (e.g., better internet connection and hardware support).

Existing roads could also be improved through better road design features such as pedestrian, livestock, and wild animal overpasses or underpasses. Corridors can facilitate wildlife movement and reduce roadkill incidents, along with signboards alerting traffic to be vigilant and drive carefully. Thorough cost-benefit analyses that consider the immediate and long-term economic, social and environmental impacts can help make informed decisions and prepare for potential challenges. An example of this is the Mombasa Road Wildlife Corridor that traverses 2.3 km of road and stretches from Tsavo East National Park boundary in the north with Jojoba, Rukinga, and Wangalla Ranches in the south of the park in Kenya.^34^

Well-marked crosswalks, designated cycling lanes, speed bumps, road resurfacing, and traffic-calming measures in residential areas, adequate lighting, visibility, signage, and road markings can significantly enhance safety for all users.

Implementing stricter vehicle emission standards and promoting the use of cleaner fuels can significantly reduce air pollution. Utilizing road materials with higher albedo (reflectivity) can reduce the heat absorption of roads. Light-colored pavements or special coatings that reflect more sunlight can help mitigate the increasing temperatures on land. The reuse of waste materials for road construction has been suggested as an alternative to waste disposal and reducing impacts of other traditional road materials^115^ but come with the risk of becoming additional sources for pollution. Integrating green spaces, such as trees, shrubs, and grassland areas, alongside roads can help counteract the heat island effect and help maintain local microclimates.^116^ Permeable pavements and water retention and drainage systems for roads and sidewalks allow water to percolate through the surface, reducing runoff and helping to maintain soil moisture and humidity levels.^117^ Vegetative buffers alongside roads can help to mitigate wind and noise disturbances and filter pollutants.^118^

Despite the significant impact that these innovations and measures may have in mitigating negative road effects, they are unlikely to be sufficient to minimize all negative road impacts and to harness the full potential positive impacts for all stakeholder groups equally. This requires a more fundamental shift in paradigms and visions behind road development (cf. solution E).

#### Road planning should rely more on evidence-based theories of change and less on universal development paradigms

Building safe and lasting road infrastructure requires substantial investments and can have many positive, but also negative consequences for socio-economic development and the environment (cf. challenges no. 1, 2, and 3). Corresponding targets have been included explicitly in the Sustainable Development Goals (SDGs 9 and 11).

Impacts of roads are also inherently context dependent, which often renders universal development paradigms useless as a basis for planning road infrastructure investments at country and sub-national scales (cf. challenge no. 3). This calls for a genuinely evidence-based approach to developing context-adapted theories of change as an input to road investment planning. Such theories of change systematically build on existing evidence and are expanded or revised as new data become available and evidence emerges^119^ (cf. solutions B and C). Many dimensions of road impacts have been evaluated using rigorous methods, but often gaps in the evidence prevent us from transferring knowledge from one context to another. A first step toward assessing the available evidence is the elaboration of evidence gap maps. In the context of road investments, this has recently been achieved in the field of road safety interventions.^120^ Fatal road accidents are notoriously high in sub-Saharan Africa, with recent reductions driven only by very few countries, such as South Africa and Nigeria.^67^

Evidence on impacts of roads on the environment, but also many relevant development outcomes, remains patchy especially for the African continent. The existing evidence, however, can be used to inform context-specific theories of change if decision-makers embraced participatory approaches to road infrastructure planning. Involving diverse stakeholders, such as local communities (including indigenous groups), civil society, and the private sector at relevant stages in road infrastructure development plans can help to complement a patchy evidence base in road planning processes and to consider more diverse stakeholder perspectives and needs (cf. challenge no. 5).

Importantly, a cross-sectoral perspective is needed in the planning process to balance expected impacts across all dimensions of the SDG (cf. challenge no. 1). Following a do no-harm principle may then often require complementary safeguards and investments in other policy fields, such as the establishment of protected areas in road expansion zones to avoid environmental degradation or structural transformation support, when new road connections put elements of local economic systems at a comparative disadvantage.

Abandoning traditional development paradigms in road planning thus also fundamentally requires acknowledging evidence of undesirable road impacts and the development of appropriate countermeasures. Power asymmetries among the stakeholders involved in and affected by road planning, may then often imply the need to create an institutional environment that ensures equal consideration of all stakeholders’ needs and interests (cf. challenge no. 5).

## Discussion and outlook

In this article, we provide an overview of five core messages about road impact research and distill five recommendations for researchers and decision-makers in road development. Siloed research approaches are insufficient to capture the complex road effects that vary across life stages, with road types and across geographic context. As this complexity is often not sufficiently considered in decision-making and road investments and planning are highly influenced by power dynamics, we often find disparities between visions and materialized impacts of roads. Consequently, scientists and decision-makers need to make use of interdisciplinary approaches that utilize innovative data collection and analysis approaches considering all life cycle stages. Apart from the utilization of social and technological innovations to reduce negative impacts, road planning should follow evidence-based theories of change rather than conventional development paradigms.

This paper also underscores the imperative of evidence-based and participatory governance mechanisms in guiding road development. Relying on empirical data and involving local communities in decision-making processes, would provide a basis for a better alignment of infrastructure projects with the needs and aspirations of the affected regions. A collaborative governance approach to road development involving multiple stakeholders can ensure that voices of affected populations are considered resulting in more inclusive outcomes. This approach would not only enhance the efficacy of road networks but also support a more equitable distribution of benefits. This requires local institutions that enable effective public participation in planning processes and an evaluation of costs and benefits of road investments as well as associated loans from third parties and countries. Novel financing models that tie investments to measurable and regularly monitored outcomes (e.g., economic growth and reduced inequality) as well as minimized environmental impacts throughout the life cycle of a road (e.g., measures to reduce deforestation along roads) can help to reduce unintended negative impacts of road development.

Furthermore, it requires novel methodological developments that are not only implemented by academics but also utilized in existing or new policy measures such as mandatory, holistic and long-term ESIAs assessments. The Green Highways Mission (India), Climate Adaptation in Africa’s Transport Sector (CAATS), and the Roads and Biodiversity Initiative (ROBIO) are example initiatives showing how road infrastructure can balance development with environmental and social considerations. These initiatives are examples how to integrate afforestation, climate resilience, and biodiversity preservation into road planning while fostering local community engagement and disaster preparedness. They highlight the transformative potential of sustainable road development in addressing ecological and social challenges in the Global South but also tend to focus on single isolated aspects rather than addressing multiple challenges at the same time.

Based on these considerations we recommend the following guiding principles for future road development efforts.

(1) Foster collaborative road governance. Governments and international investors can increase their efforts to support and implement collaborative governance approaches in road development planning ensuring voices of different stakeholder groups are sufficiently considered and heard.
(2) Leverage novel technologies, data, and tools. Advanced technologies such as AI and satellite images should be used to monitor road impacts, predict socio-economic outcomes and prevent unintended negative impacts e.g., on biodiversity.
(3) Strengthening of local institutions. Strengthening existing governance mechanisms and tools such as ESIAs and providing access to legal justice for all groups of society during road development is crucial to ensure a more equal distribution of benefits from road development.
(4) Innovate financing mechanisms. Finding other means to develop infrastructure than mere reliance on international donors and alignment of these mechanism measurable and monitored sustainability goals can increase the accountability of road developers and governments.
(5) Reframing narratives. Moving away from the traditional development narrative where roads are seen as pure drivers to an honest debate about positive and negative impacts e.g., in policy frameworks and election campaigns requires a shift in mindsets and values that can only be achieved through changing individual and collective attitudes and values.

### Limitations of the study

This study relied mainly on the insights of individual case studies and findings gathered by a large group of authors from the Global North and Sub-Saharan Africa. While we aim to provide a comprehensive objective assessment of the current challenges related to road governance and research as well as potential solutions to deal with them, we cannot rule out that our perspective is tainted by our positionalities and experiences.

## Resource availability

### Lead contact

Lisa Biber-Freudenberger (lfreuden@uni-bonn.de) is the lead contact and takes full responsibility for this role.

### Materials availability

This study did not generate new unique materials.

### Data and code availability

The datasets used in this study for visualization purposes are publicly available datasets that can be accessed under the links provided in the references.

## Acknowledgments

We are grateful for the valuable comments provided by the reviewers as well as the editor. This study was supported by the CRC Future Rural Africa (CRC228) funded by the German Research Foundation. L.B.-F. and P.J.M. also acknowledge funding provided through the German Ministry for Education and Research (BMBF) under the LANd Use SYNergies and CONflicts (LANUSYNCON) project (grant number 01UU2002).

## Author contributions

This paper is a collaborative effort from a large number of authors of which all contributed to editing and improving the text of this manuscript. L.B.-F., C.B., J.P.R.T., and J.B. conceptualized the study; L.B.-F., C.B., T.K., V.M., N.N., J.P.R.T., and J.B. wrote different parts of the original paper draft; G.B., M.B., P.D., J.R.D., C.G., B.K., D.M.-M., D.O.O., E.K., and T.T. reviewed and edited the original draft. L.B.-F. coordinated the writing and publication process.

## Declaration of interests

The authors declare no competing interests.

## Declaration of generative AI and AI-assisted technologies in the writing process

During the preparation of this work the authors used ChatGPT in order to improve the flow of certain sentences. After using this tool, the authors reviewed and edited the content as needed and take full responsibility for the content of the publication.
